# When Similarity Beats Expertise—Differential Effects of Patient and Expert Ratings on Physician Choice: Field and Experimental Study

**DOI:** 10.2196/12454

**Published:** 2019-06-26

**Authors:** Anne-Madeleine Kranzbühler, Mirella H P Kleijnen, Peeter W J Verlegh, Marije Teerling

**Affiliations:** 1 Department of Product Innovation Management Delft University of Technology Delft Netherlands; 2 Department of Marketing Vrije Universiteit Amsterdam Amsterdam Netherlands; 3 Eneco Rotterdam Netherlands

**Keywords:** decision making, choice behavior, judgment

## Abstract

**Background:**

Increasing numbers of patients consult Web-based rating platforms before making health care decisions. These platforms often provide ratings from other patients, reflecting their subjective experience. However, patients often lack the knowledge to be able to judge the objective quality of health services. To account for this potential bias, many rating platforms complement patient ratings with more objective expert ratings, which can lead to conflicting signals as these different types of evaluations are not always aligned.

**Objective:**

This study aimed to fill the gap on how consumers combine information from 2 different sources—patients or experts—to form opinions and make purchase decisions in a health care context. More specifically, we assessed prospective patients’ decision making when considering both types of ratings *simultaneously* on a Web-based rating platform. In addition, we examined how the influence of patient and expert ratings is conditional upon rating volume (ie, the number of patient opinions).

**Methods:**

In a field study, we analyzed a dataset from a Web-based physician rating platform containing clickstream data for more than 5000 US doctors. We complemented this with an experimental lab study consisting of a sample of 112 students from a Dutch university. The average age was 23.1 years, and 60.7% (68/112) of the respondents were female.

**Results:**

The field data illustrated the moderating effect of rating volume. If the patient advice was based on small numbers, prospective patients tended to base their selection of a physician on expert rather than patient advice (profile clicks beta=.14, *P*<.001; call clicks beta=.28, *P*=.03). However, when the group of patients substantially grew in size, prospective patients started to rely on patients rather than the expert (profile clicks beta=.23, SE=0.07, *P*=.004; call clicks beta=.43, SE=0.32, *P*=.10). The experimental study replicated and validated these findings for conflicting patient versus expert advice in a controlled setting. When patient ratings were aggregated from a high number of opinions, prospective patients’ evaluations were affected more strongly by patient than expert advice (mean_patient positive/expert negative_=3.06, SD=0.94; mean_expert positive/patient negative_=2.55, SD=0.89; *F*_1,108_=4.93, *P*=.03). Conversely, when patient ratings were aggregated from a low volume, participants were affected more strongly by expert compared with patient advice (mean_patient positive/expert negative_=2.36, SD=0.76; mean_expert positive/patient negative_=3.01, SD=0.81; *F*_1,108_=8.42, *P*=.004). This effect occurred despite the fact that they considered the patients to be less knowledgeable than experts.

**Conclusions:**

When confronted with information from both sources simultaneously, prospective patients are influenced more strongly by other patients. This effect reverses when the patient rating has been aggregated from a (very) small number of individual opinions. This has important implications for how to present health care provider ratings to prospective patients to aid their decision-making process.

## Introduction

### Background

The rise of the internet, with its abundance of information, has changed human decision making [1,2]. To reduce their uncertainty when facing a choice, people have always sought advice from other people [3-5]. Web-based information sources that provide electronic word of mouth have become ubiquitous, which has also influenced people’s health care decisions [6-8]. When choosing a physician, for example, many patients consult health care rating platforms to obtain information about the quality of different providers [9,10]. Such platforms may be provided by governments (eg, United States: medicare.gov, United Kingdom: nhs.uk) or companies (eg, healthgrades.com, betterdoctor.com) and allow people to consult other patients’ ratings of institutions, physicians, or specific procedures [11]. Parallel to this increased use of ratings by consumers, these ratings have also gained importance in public health systems; for example, in the United States [12,13]. Medicare social insurance program financially penalizes hospitals that perform poorly in their patient ratings [14,15].

Such uses of patient ratings might be questionable though, because health care, similar to other professional services, is complex and marked by credence qualities [16,17]. Even after it has been performed, it is difficult for patients to assess the actual quality of a health service, especially because laypeople often lack the skills needed to evaluate the complex offerings [18]. Accordingly, when prior patients evaluate a physician on a Web-based rating platform, they tend to highlight easily observable features related to the care, such as a physician’s convenient location or friendly office staff [7,19], rather than cure-related outcomes [20]. A positive service experience is integral to health care, in that it contributes to psychological and physiological health [21], but such assessments ignore another essential element: the technical quality of health care procedures [13]. Moreover, though consumers often use their service experience as a proxy for technical quality, these factors are at best related, not substitutive, components of the same assessment [22,23]. To address this problem, many rating platforms include outcome-focused expert judgments based on statistics, such as readmission rates, as complementary sources of information. However, little is known about how consumers combine information from the 2 different sources—namely, similar patients or experts (with detailed knowledge)—to form opinions and make purchase decisions.

This study aims to fill this gap. On the basis of source effects literature, we investigated prospective patients’ decision making when simultaneously exposed to other patients’ and expert ratings on Web-based platforms. Whereas previous research primarily focused on the effects of *either* user or expert ratings on decision making in various contexts (eg, as seen in some studies [24-27]), we focused on situations where both types of ratings are provided *simultaneously*. This design feature of Web-based rating platforms is not only of increasing practical interest, but it also allows for disagreement between the patient and expert advice, which is of theoretical interest because it forces prospective patients to make a decision about which source they want to follow. The literature on source effects suggests that patients may overly rely on information from similar sources, so that patients may base their decisions more strongly on advice from other patients (who are more similar to them) than from experts, although the latter may be seen as more competent to give advice in the respective situation [27].

We further investigated how the influence of patient and expert ratings is dependent on the number of patient opinions that contributed to the aggregated patient rating (hereafter referred to as “rating volume”). Rating volume has been widely studied in the literature on Web-based reviews and ratings (eg, [11,28-30]), but little is known about how website users make use of this additional cue when simultaneously confronted with different sources of advice. A common design practice of Web-based rating platforms is to present only 1 expert rating, whereas the patient rating typically is based on the aggregated opinion of multiple patients. On the basis of social influence theory, we argue that the influence of patient over expert ratings is dependent on the number of underlying patient opinions that contributed to the overall rating. This is not only an important insight for practitioners who have to decide about the design of such a platform, but it also helps to understand the psychological processes underlying patients’ preferences for advice from other patients or experts.

### Source Effects

To determine the usefulness of a recommendation, a receiver must evaluate its source [31]. A source’s perceived expertise and perceived similarity to the receiver offer the best predictors of its impact on decision making [32,33]. Although other patients can be expected to be perceived as more similar than experts, they most likely lack the experts’ detailed knowledge.

#### Similarity

Source similarity describes the extent to which people resemble one another on certain attributes. Demographic similarity refers to attributes such as gender, age, or socioeconomic status; attitudinal similarity instead pertains to values, attitudes, or lifestyles [32]. Similar sources tend to be particularly influential because they share similar needs and preferences with the receiver and thus deliver relevant information [34,35]. Similarity also leads to greater attractiveness, trust, and understanding [36], which facilitates communication [37]. Therefore, information from similar others is more persuasive and has a stronger influence on decision making than information from dissimilar others (eg, [35,38]). However, socially meaningful similarities can create a perceived bond between people, in addition to the fact that they share a similar experience or situation [39]. In the present health care context, the user might realize that other patients have been in the same situation of needing a certain treatment. Experts instead might be perceived as being less similar as they do not share the same situation and probably approach the assessment of a service in a more abstract and technical manner.

#### Expertise

Expertise is the source’s “ability to perform product-related tasks successfully” [40]. Perceptions of a source’s expertise stem from evaluations of its knowledge, experience, or occupation [41]. In a Web-based environment, this evaluation relies on the limited available cues [42], although most consumers accept an expert source presented on a website and do not investigate the basis of its expertise further [43,44]. The influence of expert sources stems from their expansive knowledge base, compared with nonexpert sources [32,45]. They have greater knowledge about various, alternative offerings because of their enduring involvement in a certain product or service category [46]. In turn, experts’ opinions appear to be of high quality, and their advice has a strong, positive impact on receivers (eg, [32,47,48]).

#### Comparing the Impact of Similarity Versus Expertise

Both perceived expertise and perceived similarity enhance the persuasiveness of a source (eg, [49]), but we do not know which source has the stronger influence: the expert or the similar peer. Research so far compares their impact only indirectly, by employing survey methods or confronting participants with either type. An early study found that consumers are more inclined to follow the advice of a similar but inexperienced salesperson compared with a dissimilar but experienced one [34]. In line with this, expert reviews have been found to reduce consumers’ willingness to visit a restaurant’s website, whereas consumer reviews increase it [50]. Similarly, when looking for a new doctor, most participants in a US study sought advice from similar sources rather than those with medical expertise [51]. Yet, other studies indicate instead that source expertise is a better predictor of influence [32,52]. A recent meta-analysis affirms a greater impact on sales elasticities from a review provided by an expert rather than a consumer source [53]. Thus, there is conflicting evidence from various contexts, which leads to our main research question:

What is the impact of expert and patient ratings when simultaneously provided on a Web-based rating platform?

#### The Moderating Role of Rating Volume Information

In the present context of a *simultaneous* presentation of patient and expert ratings, website users might look for additional cues, such as volume information, to help them make inferences about the sources [54,55]. Rating platforms very often not only simultaneously provide star ratings from experts and patients but also inform website users about how many individual patient opinions have been aggregated to compute a rating. However, this ancillary information is usually only given for patient ratings, whereas expert ratings are not further qualified by specific volume information (eg, betterdoctor.com, nhs.uk, and independer.nl). Thus, this additional cue might impact the way website users construe the patient rating in relation to the simultaneously provided expert rating. This design feature leads to the theoretically profound question—whether information about rating volume (ie, the number of opinions of other patients that underlie a physician rating) may constitute an important moderator for the influence of such ratings on other patients’ service evaluations.

Social influence literature states that the likelihood that an individual imitates a certain behavior increases with the number of other individuals who display this behavior [56,57]. The high number of such individuals increases the perceived benefits of adopting a certain behavior compared with its perceived costs [58,59]. Numerical information has been found to be automatically encoded by the human brain and thus easy to process relatively independent of individual differences [60]. The numerical dominance of a majority opinion signals its correctness [61,62]. Humans have been found to be especially prone to the influence from a large number of others when it comes to making purchase decisions (eg, [63-65]). Humans engage in such imitative behavior because a high volume increases the diagnosticity of a message’s valence and thus its persuasiveness [29]. This might be due to the fact that high consensus signals that those others might possess a piece of information that is not yet available to the decision maker, (ie, “they must all know something I don’t”) [63,66]. In line with that, the credibility as well as the impact of a group’s judgment on an individual have been found to be positively correlated with the group’s size—both Web-based and offline (eg, [28,67]). Hence, rating volume is likely to amplify the effect of advice from similar patients (compared with expert advice) on physician evaluation and decision making. In other words: the larger the number of patients who rate a physician, the more influential the (averaged) patient rating. We thus hypothesize the following:

The influence of patient (versus expert) ratings on(1) evaluations and (2) usage intentions of a health service is moderated by rating volume, such that with increasing numbers of underlying patient opinions, the influence of the patient over the expert rating increases.

## Methods

### Study 1: Field Study

Study 1 relies on clickstream data from an actual US health care rating platform to assess the impact of simultaneously provided patient and expert ratings as well as the moderating role of explicit rating volume information. The US-based rating platform provides expert and patient ratings on more than 1 million doctors, dentists, and eye doctors throughout the United States to help patients find a suitable service provider. For each doctor, an algorithm calculates an expert rating on the basis of the physician’s education, experience, training, and referrals by other doctors. It also displays the average Yelp (patient) rating for each doctor. Both rating types are presented next to each other on overview and search results pages from which patients can access the individual doctor profiles. We assessed the impact of both rating types on 2 behavioral actions: the number of times website users viewed a certain doctor’s profile and the number of times they clicked a button to obtain the doctor’s contact information.

#### Data Collection

We obtained clickstream data for 5299 doctors during May 1 to July 31, 2015 from the rating platform’s Web analytics tool and aggregated the click-level data (21,897 profile clicks and 1842 call clicks, see below) to the doctor-level (n=5299). We only included doctors whose profiles featured both expert and patient ratings and whose profiles had been viewed at least once by a prospective patient during the data collection period. We extracted how many times each doctor’s profile had been viewed and how many times users clicked to obtain the doctor’s contact information. Furthermore, for each doctor, we extracted the expert star rating, the Yelp (patient) star rating, and the rating volume; the overall Yelp rating was based on at the moment of the click. In addition, we collected several control variables (see below).

#### Measures

We have 2 dependent variables: number of profile clicks and number of call clicks, that is, we counted the number of times each doctor profile was viewed by prospective patients in the data collection period. We also determined how many times visitors to a specific doctor’s profile clicked on a button to obtain this doctor’s phone number. This behavioral measure provides a proxy for doctor choice.

The independent variables reflected expert and patient ratings. Expert ratings were the professional star ratings provided for each doctor, which ranged from 1 to 5 in 0.5 steps (half stars). The average expert rating was very positive (mean 4.27, SD 0.94). Patient ratings were provided in the form of Yelp star ratings on each doctor’s profile. The Yelp ratings could change during the data collection period; we therefore used the respective Yelp rating at the moment of each individual click to calculate an average patient rating for each doctor over the data collection period. Patient ratings also varied from 1 to 5, in 0.5 steps, and the average patient rating in our sample was also positive (mean 3.89, SD 1.21). To be able to test the effect of rating volume, we included the number of individual patient opinions the Yelp star rating was based on in our model. This number could also change over the course of the data collection period; therefore, we calculated an average rating volume for each doctor again (the correlations can be found in Multimedia Appendix 1).

Finally, to isolate the effects of expert and patient ratings on the click-based dependent variables, we included doctor-specific control variables. We controlled for the number of doctor referrals. On the basis of 160 million Medicare referrals from 2009 to 2012, this number indicated how many patients were referred by other doctors to a specific doctor. Doctor referrals only appear on a profile when they are greater than 0, so we included a dummy variable for whether doctors had any referrals or not (0=no, 1=yes). Furthermore, we controlled for each doctor’s specialty; the number of practices in which she or he is employed; and whether the doctor has a profile image (0=no, 1=yes), photo gallery (0=no, 1=yes), or Web-based booking system incorporated in the profile (0=no, 1=yes), as well as whether a doctor has a premium profile (0=no, 1=yes) on the platform.

#### Model Specification

Most doctors in our sample received few profile or call clicks during the data collection period, and a few doctors had many visitors. Thus, both dependent count variables are overdispersed (mean_profile clicks_ 4.12, variance 85.89; mean_call clicks_ 0.34, variance 3.03), and we therefore modeled both dependent variables with a negative binomial regression [68]. To account for systematic differences between different kinds of doctors, we included a random intercept for each of the 57 doctor specialties:

log(profile clicks_i_) = α_0_ + α_j_ + β_1_(expert rating_i_) + β_2_(consumer rating_i_) + β_3_(consumer rating_i_ × rating volume_i_) + ΩX_i_ + ε_i_,

where i indexes the doctor and j the doctor specialty, X_i_ is the vector of doctor-specific control variables, and ε_i_ is the error. The model specification for our second dependent measure, call clicks, is analogous. For our call clicks model, we controlled for all variables; for the profile clicks model, we only controlled for doctor specialty, practice count, profile image, and premium profile, as the other control variables only become visible to consumers once they have clicked on a doctor’s profile (and not on the search results page).

Although the analysis of real clickstream data is useful for getting first insights into the effect of simultaneously provided expert and patient ratings, these are only descriptive in nature. As we are interested in the causal process, we complement our first study with an experimental study in a controlled setting. In study 2, we systematically manipulated expert and patient ratings as well as rating volume. As such, study 2 aimed at finding further support for the causal influence of the number of underlying patient opinions (H1) by replicating the findings from study 1 in an experimental setting.

### Study 2: Experimental Study

#### Procedure and Sample

We employed a 2×2 between-subjects design, in which we manipulated the valences of the expert and patient rating (positive vs negative) and the number of individual opinions the patient rating is based on (high volume vs low volume). We included only manipulations of conflicting ratings (ie, expert positive and patient negative and vice versa-we refer to this variable as “positive source type,” as it can be “patient positive” or “expert positive”). Our objective was to compare a very high and a very low rating volume to be able to effectively contrast the respective effects. Drawing from prior research [64], observations of a Dutch health care rating platform (independer.nl), and especially from our study 1 dataset, we set the high rating volume to 142 (among top 1% of number of underlying opinions in our dataset). We set the low rating volume to 3 so that the aggregated patient rating resembled more than just a single but still only very few opinions. We recruited 125 undergraduate students from a Dutch university in exchange for course credit and randomly assigned them to 1 of the 4 experimental conditions in a computer lab. We excluded 13 participants who did not recall that there was a patient and expert star rating on the site. In our sample (n=112), participants had a mean age of 23.1 years, and 60.7% (68/112) were female.

Participants imagined that they were looking for a hospital to perform a surgery, so they consulted a rating platform to help make their decision (the manipulation can be found in Multimedia Appendix 2). On the following page, the evaluation of a fictional hospital, on a fictional health care rating platform, featured both an expert and a patient star rating. Those ratings were conflicting and either negative (1.5 out of 5 stars) or positive (4.5 out of 5 stars). The patient rating was either based on 3 or 142 individual opinions. Next, participants indicated their attitudes toward the hospital [69,70] and their usage intentions [71]. These variables correlated (*r*=.83, *P*<.01), so we combined them into a single evaluative index (“hospital evaluation”; alpha=.96). We also measured participants’ evaluations of experts and patients with regard to their expertise (experts: alpha=.94; consumers: alpha=.83 [72]) and trustworthiness (experts: alpha=.95; consumers: alpha=.88 [73]). All these measures featured 5-point scales (the correlations can be found in Multimedia Appendix 1; the reliability of scales are presented in Multimedia Appendix 3).

## Results

### Study 1: Field Study

Table 1 shows the results of the regression models. The analyses for the profile clicks and call clicks models exclude 5 doctors (profile clicks model) and 1213 doctors (call clicks model) because of missing values for the predictors, resulting in a sample size of 5294 for the profile clicks model and 4086 for the call clicks model. In our 2 initial models (models 1 and 3), we found a significant positive effect of the expert rating on both profile clicks (beta=.13, *P*<.001) and call clicks (beta=.32, *P*<.001) and of the patient rating on profile clicks (beta=.03, *P*=.02) but not on call clicks (beta=.04, *P*=.76). The interactions of patient rating and rating volume (mean centered [74]) on profile clicks (beta=.01, *P*<.001) and call clicks (beta=.01, *P*<.001) were, however, significant. To investigate these interaction effects further and assess the effect of the patient rating on the dependent variables with different numbers of underlying individual ratings, we repeated the analyses with a mean-centered value for the rating volume plus 3 SDs (models 2 and 4). As expected, the effect of the patient rating increased with the number of individual reviews aggregated to create the rating. As we noted previously, the effects of patient ratings were only significant for profile but not for call clicks for an average rating volume (Yelp rating based on 9 opinions), but their effects were significant and comparable with those of expert ratings when the underlying number of patient opinions was 1 SD higher (Yelp rating based on 27 opinions; profile clicks beta=.14, *P*<.001; call clicks beta=.28, *P*=.03). The effects of expert and patient ratings were not significantly different from each other: profile clicks beta=.01, SE 0.04, *P*=.37; call clicks beta=−.05, SE 0.15, *P*=.38. When the patient rating was based on the average rating volume plus 3 SDs (Yelp rating based on 63 opinions), the effects on both dependent variables (profile clicks beta=.35, *P*<.001; call clicks beta=.75, *P*=.02) were even stronger. With the number of underlying patient opinions increasing to 63, the effect of the patient rating becomes (marginally) significantly stronger than the effect of the expert rating (difference profile clicks beta=.23, SE 0.07, *P*=.004; call clicks *β*=.43, SE 0.32, *P*=.10).

The results of the analysis of field data thus confirm rating volume (ie, the number of underlying patient reviews) to be an important moderator of the impact of patient versus expert advice. Specifically, we find that when confronted with both sources simultaneously, prospective patients tend to base their evaluations of a physician on expert rather than patient advice in case the patient advice is based on small numbers. However, when the group of patients substantially grows in size, prospective patients start to rely more on patients rather than the expert in making a decision, which supports H1. Thus, patients are indeed influenced more strongly by advice from other patients than by advice from experts, but this occurs only when the patient advice is based on a large number of individual opinions. This finding is in line with prior research stating that volume increases the perceived diagnosticity and the effect of Web-based reviews (eg, [28,29]).

**Table 1:** Regression results of study 1.

Independent variables		Dependent variable: log profile clicks				Dependent variable: log call clicks			
		Model 1		Model 2		Model 3		Model 4	
		Coefficient	SE	Coefficient	SE	Coefficient	SE	Coefficient	SE
Expert rating		0.13^a^	0.02	0.13^a^	0.02	0.32^a^	0.06	0.32^a^	0.06
Patient rating × rating volume (centered at mean)		0.01^a^	0.00	—^b^	—	0.01^a^	0.01	—	—
Patient rating (at mean level of rating volume)		0.03^c^	0.01	—	—	0.04^a^	0.05	—	—
Rating volume		0.00^a^	0.00	—	—	−0.00^a^	0.00	—	—
Patient rating × rating volume (centered at mean + 3 SD)		—	—	0.01^a^	0.00	—	—	0.01^a^	0.01
Patient rating (at mean level of rating volume + 3 SD)		—	—	0.35^a^	0.07	—	—	0.75^a^	0.31
Rating volume		—	—	0.00^a^	0.00	—	—	−0.01^a^	0.00
**Controls**									
	Doctor referrals	—	—	N/A	—	−0.00^a^	0.11	−0.00^a^	0.11
	Doctor referrals count	—	—	N/A	—	0.00^b^	0.00	0.00^a^	0.00
	Practice count	0.00^a^	0.01	0.00^b^	0.01	0.07^a^	0.02	0.07^a^	0.02
	Profile image	0.03^a^	0.03	0.03^b^	0.03	−0.07^a^	0.10	−0.07^a^	0.10
	Photo gallery	—	—	—	—	0.64^a^	0.15	0.64^a^	0.15
	Web-based booking	0.19^a^	0.03	0.19^a^	0.03	0.05^a^	0.11	0.05^a^	0.11
	Premium profile	2.03^a^	0.12	2.03^a^	0.12	0.98^a^	0.44	0.98^a^	0.44
Intercept		0.72^a^	0.07	84^a^	0.09	−3.34^a^	0.29	−3.45^a^	0.30
Log-likelihood		−12910.8	—	—	—	−2257.7	—	—	—
Akaike information criterion		25843.5	—	—	—	4543.4	—	—	—

^a^Significant at the *P*<.01 level.

^b^Not applicable.

^c^Significant at the *P*<.05 level.

### Study 2: Experimental Study

To check whether participants indeed perceived the experts and patients in our stimuli as such, we investigated whether expert and patient ratings were perceived differently with regard to expertise and trustworthiness, and whether this depended on the additional rating volume information. Experts were generally perceived as higher in expertise (mean 3.95, SD 0.73) than patients (mean 2.57, SD 0.69; *F*_1,110_=190.33, *P*<.001), which was independent of the number of underlying patient opinions in the experimental condition (*P*>.85). Trustworthiness did not differ between experts and patients, again independent of the number of underlying patient opinions (all *P* values>.20).

**Figure 1:**
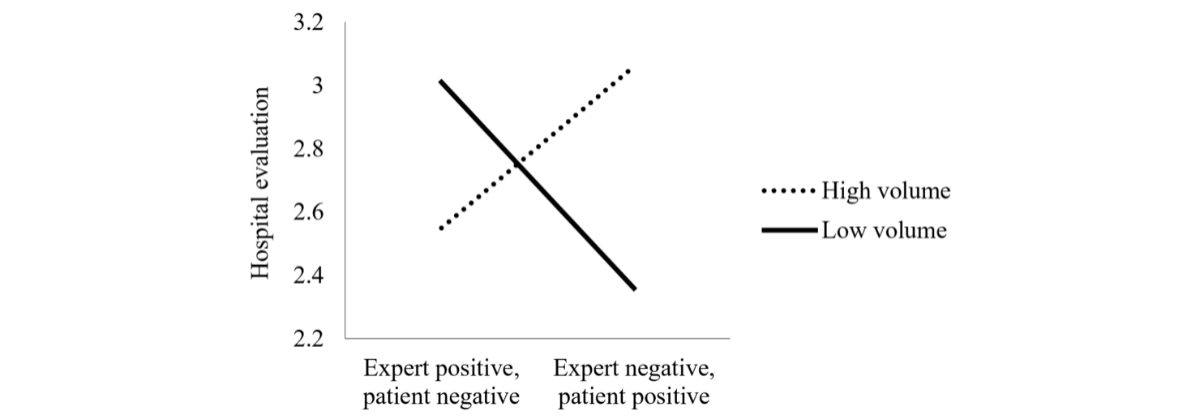
Study 2: Interaction effect of positive source type and rating volume on hospital evaluation.

The results of an analysis of variance indicated no significant main effects of positive source type (patient positive vs expert positive) and rating volume (142 vs 3) on hospital evaluation (*F*_positive source type__1,108_=0.17, *P*=.68; *F*_volume__1,108_=0.52, *P*=.47). However, as expected, we found a significant interaction effect of these 2 variables on hospital evaluation (*F*_1,108_=13.06, *P*<.001). Further analyses demonstrated that when patient ratings are aggregated from a high number of opinions, prospective patients’ evaluations are affected more strongly by patient than expert advice (mean_patient positive/expert negative_ 3.06, SD 0.94; mean_expert positive/patient negative_ 2.55, SD 0.89; *F*_1,108_=4.93, *P*=.03). Conversely, when patient ratings are aggregated from a low volume, participants are affected more strongly by expert compared with patient advice (mean_patient positive/expert negative_ 2.36, SD 0.76; mean_expert positive/patient negative_ 3.01, SD 0.81; *F*_1,108_=8.42, *P*=.004; Figure 1). Thus, study 2 finds further support for H1.

The results of study 2 thus further support the findings from study 1: prospective patients are influenced more strongly by other patients when the patient evaluation is based on a larger number of individual opinions, but not when it is based on only a few observations. When the patient rating has been aggregated from only a small number of individual opinions, website users are instead more inclined to follow the expert advice. However, we find that participants still perceive experts to be higher in expertise, even than a large number of other patients. Moreover, and in contrast to prior findings [75], website users do not seem to trust experts less than other patients or assume any biases or ulterior motives. Consequently, prospective patients seem to be aware of the fact that a large number of other patients still lack the expertise to judge the objective outcome quality of a health service and also do not trust them more than an expert giving a rating. Yet, they are more inclined to follow their peers’ compared with an expert’s advice when choosing a health service.

## Discussion

### Principal Findings

This study examined how the evaluations and usage intentions of a health service is influenced by the simultaneous exposure to conflicting patient versus expert ratings and how this relationship is moderated by rating volume. The findings support the hypothesis that with increasing numbers of underlying patient opinions, the influence of the patient over the expert rating increases. In case the patient advice is based on small numbers, prospective patients tend to base their evaluations on an expert rather than patient advice. However, when the group of patients substantially grows in size, prospective patients start to rely more on patients rather than experts in making a decision. These patients are considered to be more similar yet less knowledgeable than experts. Thus, in line with social influence and source effects literature, patients are influenced more strongly by advice from other patients than by advice from experts but only when the patient advice is based on a large number of individual opinions. This has important implications for practitioners who design health care rating platforms. These implications hold special importance for how and which ratings to present to prospective patients to aid their decision-making process. It further helps us to understand the psychological processes underlying patients’ preferences for advice from other patients or experts.

To the best of our knowledge, this study is the first to analyze the impact of the *simultaneous* presence of conflicting ratings from patient and expert sources on patient decision making. Research that provides participants with 1 source offers conflicting evidence for the preference for peer over expert advice (eg, [28,50]), but such studies cannot explicate the disaggregated effects of advice from different sources on a receiver or how consumers decide “which electronic word-of-mouth messages to adopt and which ones to reject” [76]. In 1 field study and 1 experimental study, we shed light on the differential effects of patient and expert advice in a health care context.

### Limitations

In providing initial insights into the use of conflicting advice from different sources in patient decision-making, this study features several limitations that provide directions for further research. First, in our experimental study, we did not specify who the “experts” were. Prior research indicates that users mainly look for authority cues when assessing a website’s credibility, but they rarely investigate who the expert sources actually are [44]. The participants in our studies perceived the experts as such. However, it would be interesting to explore whether the findings change when the advice comes from various experts, such as family doctors or specialist physicians.

Second, our findings only lay the foundation for understanding how users make a decision when confronted with different sources of information simultaneously. Although our analysis of clickstream data is high in external validity, it only produces correlational results. The sample was further not taken at random and should thus be interpreted with caution. Study 2 can likewise not quantify the exact effects of expert and patient ratings. Future research should investigate this further by making users choose between different offerings. Doing so will enable us to quantify the relative importance of different pieces of information.

Third, we did not analyze the potential moderating roles of other rating and platform characteristics. Platforms tend to feature both numerical ratings and textual reviews [77]. Other important characteristics, including language use, salience of the valence, or further information about the sources and their reputation [76] thus deserve further inquiry.

Fourth, this study investigated Web-based ratings, one of many potential information sources a consumer employs to make health service decisions. Offline sources such as friends and family also strongly influence health service choice (eg, [78]). Further research might examine the differential effects of offline versus Web-based word of mouth and their interaction across the different stages of the decision-making process.

### Conclusions

First, this study enhances the understanding of patients’ use of Web-based decision support tools. More specifically, we shed light on the integration of simultaneously provided information from other patients and experts. We found that the opinion of a moderate number of other patients strongly influenced users’ evaluations and choices of physicians, even overruling the conflicting opinions of experts. In this sense, choosing a health care provider does not seem to differ much from purchase choices for (for example) movies or restaurants. Even for credence services such as health care, others’ subjective ratings of service experiences have a greater impact on decision making than more objective ratings of the service. Although the subjective patient experience is also important, it often fails to acknowledge the actual outcome quality of the service which patients have difficulties evaluating even after it has been performed [79]. These 2 aspects should be considered complementary for well-informed decisions, but our study suggests that this combination is not the route most consumers take when considering different sources of quality information on a Web-based rating platform. Even in health care contexts, users turn to consumers over expert advice, and this negligence of objective outcome quality information might lead them to make suboptimal choices, with considerable consequences for their health and society at large.

Second, for rating platform providers, our results support the trend of providing advice from both expert and patient sources. Experts and patients differ in their expertise and access to information, so they often emphasize different features in their judgments. These 2 sources of advice therefore should be regarded as complementary instead of substitutive input. Furthermore, the platform providers need to include the number of individual opinions underlying a patient rating because users might assume a rating is based on many opinions if the information is not evident. When this information is present, it acts as an important moderator, preventing an overemphasis on a collection of just a few patient ratings.

## Appendix

Multimedia Appendix 1 The correlations of study 1 and 2.

Multimedia Appendix 2 Screenshots of a fictional Web-based health care rating platform used as the manipulations in study 2.

Multimedia Appendix 3 The reliability of scales.
