# Supplementary material for: When Similarity Beats Expertise—Differential Effects of Patient and Expert Ratings on Physician Choice: Field and Experimental Study
Source: J Med Internet Res. 2019 Jun 26;21(6):e12454. doi: 10.2196/12454 (PMC6617917; doi:10.2196/12454)
Supplement: Multimedia Appendix 1 [file jmir_v21i6e12454_app1.pdf]

Multimedia Appendix 1: Correlation Tables

Study 1:

Main variables	Mean	SD	Sample size	2	3	4	5	6	7	8	9	10	11	12
1. Call clicks	0.34	1.74	5299	.80[a]	.06[a]	.02	.05[a]	−.01	−.01	.01	.05[a]	.09[a]	.03[b]	.18[a]
2. Profile clicks	4.12	9.27	5299		.08[a]	.03	.08[a]	−.00	−.01	−.01	.06[a]	.24[a]	.03[b]	.30[a]
3. Expert rating	4.27	0.94	5299			.28[a]	.14[a]	.11[a]	.03	.07[a]	.46[a]	.00	.06[a]	.04[a]
4. Patient rating (mean centered)	3.89	1.21	5299				.06[a]	−.08[a]	−.05[a]	−.01	.14[a]	−.01	−.05[a]	.03[b]
5. Rating volume (mean centered)	9.00	18.11	5299					−.08[a]	−.06[a]	−.01	.13[a]	.04[b]	−.01	.02
6. Doctor referrals	0.44	0.50	5299						.48[a]	.03[b]	.04[a]	−.05[a]	.05[a]	−.05[a]
7. Doctor referrals count	13.28	31.54	5299							.04[a]	−.02	−.03	.02	−.04[a]
8. Practice count	2.13	1.83	5299								.15[a]	−.10[a]	−.04[a]	−.05[a]
9. Profile image	0.53	0.50	5299									−.02	.04[a]	.09[a]
10. Photo gallery	0.08	0.27	4086										−.02	.20[a]
11. Online booking	0.23	0.42	5299											−.05[a]
12. Premium profile	0.01	0.09	5294											

[a]Significant at the *P*<.01 level.
[b]Significant at the *P*<.05 level.

Study 2:

Main variables	Mean	SD	Sample size	2	3	4	5
1. Evaluation hospital	2.74	0.89	112	−.01	.07	.03	.03
2. Trustworthiness expert	3.27	0.85	112		.65[a]	−.16	−.10
3. Expertise expert	3.95	0.73	112			−.04	−.09
4. Trustworthiness consumer	3.11	0.83	112				.42[a]
5. Expertise consumer	2.57	0.69	112				

[a]Significant at the *P*<.01 level.
[b]Significant at the *P*<.05 level.
