# Supplementary material for: When Similarity Beats Expertise—Differential Effects of Patient and Expert Ratings on Physician Choice: Field and Experimental Study
Source: J Med Internet Res. 2019 Jun 26;21(6):e12454. doi: 10.2196/12454 (PMC6617917; doi:10.2196/12454)
Supplement: Multimedia Appendix 2 [file jmir_v21i6e12454_app2.pdf]

Multimedia Appendix 2: Study 2

Scenario

Imagine you have been diagnosed with a serious heart condition for which it is essential that you undergo surgery. The surgery can be performed at several hospitals. You are searching for information to aid you in your decision in which hospital you want to have the surgery done. For this purpose, you are visiting an independent rating platform. On the following page you will see a screen shot of this rating platform for a fictional hospital.

Screenshot manipulation

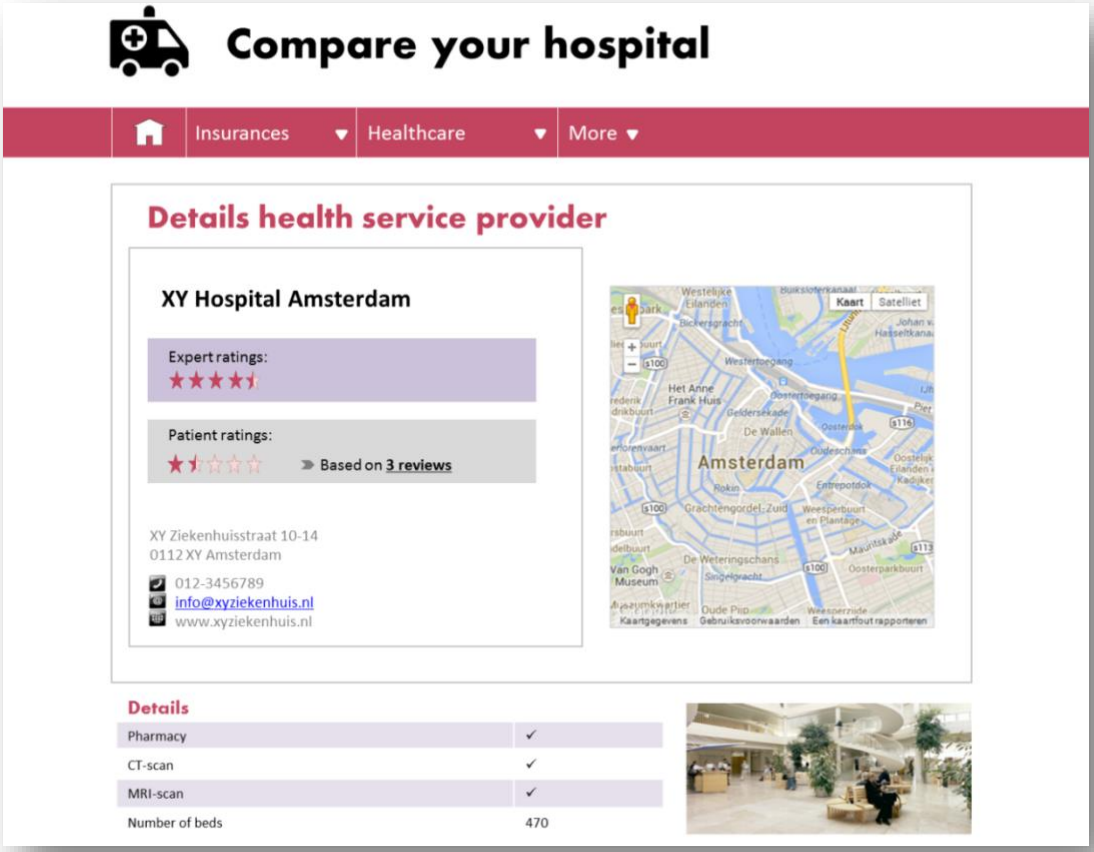

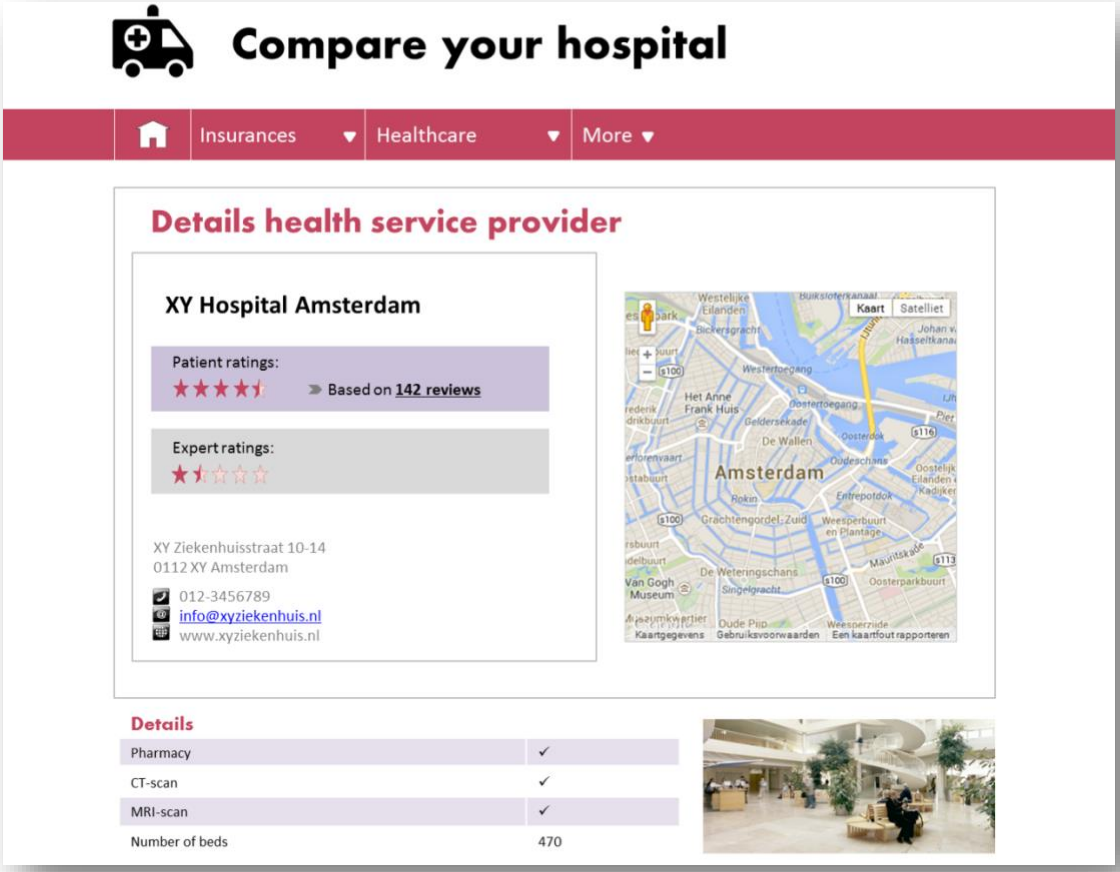
Compare your hospital
Insurances
Healthcare
More
Details health service provider
XY Hospital Amsterdam
Patient ratings:
Based on 142 reviews
Expert ratings:
XY Ziekenhuisstraat 10-14
0112 XY Amsterdam
012-3456789
info@xyziekenhuis.nl
www.xyziekenhuis.nl
Kaart
Satelliet
Amsterdam
Details
Pharmacy ✓
CT-scan ✓
MRI-scan ✓
Number of beds 470
