# Supplementary material for: When Similarity Beats Expertise—Differential Effects of Patient and Expert Ratings on Physician Choice: Field and Experimental Study
Source: J Med Internet Res. 2019 Jun 26;21(6):e12454. doi: 10.2196/12454 (PMC6617917; doi:10.2196/12454)
Supplement: Multimedia Appendix 3 [file jmir_v21i6e12454_app3.pdf]

Multimedia appendix 3: Reliability of Scales

Main constructs	*Items*	Coefficient alpha
		Study 2
(1) Hospital evaluation[a]	My overall impression of this hospital is..<br>..(good / bad)<br>..(favorable / unfavorable)<br>..(negative / positive)<br>..(unsatisfactory / satisfactory)<br>Would you go to this hospital to operate your knee?<br>..(Highly unlikely / Highly likely)<br>..(No chance at all / Very good chance)<br>..(Highly improbable / Highly probable<br>..(Highly uncertain / Highly certain	.96
Item sources: Becker-Olsen (2003); Rodgers (2003); Chandran & Morwitz (2005)		
(2) Expertise (expert source)	What is your opinion about experts who give such ratings? The experts are…<br>..(inexperienced / experienced)<br>..(unknowledgable / knowledgable)<br>..(unqualified / qualified)<br>..(unskilled / skilled)	.94
Item source: Ohanian (1990)		
(3) Expertise (consumer source)	What is your opinion about patients / consumers who give such ratings? The patients / consumers are…<br>..(inexperienced / experienced)<br>..(unknowledgable / knowledgable)<br>..(unqualified / qualified)<br>..(unskilled / skilled)	.83
Item source: Ohanian (1990)		
(4) Trustworthiness (expert source)	What is your impression of such expert ratings?<br>I trust such expert ratings.<br>Such expert ratings make truthful claims.<br>Such expert ratings are honest.<br>I believe what such expert ratings tell me.	.95

Item source: Newell & Goldsmith (2001)		
(5) Trustworthiness (consumer source)	What is your opinion of such patient / consumer ratings?<br>I trust such patient / consumer ratings.<br>Such patient / consumer ratings make truthful claims.<br>Such patient/ consumer ratings are honest.<br>I believe what such patient / consumer ratings tell me.	.88
Item source: Newell & Goldsmith (2001)		

a. Attitudes toward the hospital and usage intentions were combined into the hospital evaluation measure as both variables correlated (r = .83, $p < .01$).
